# Accessing HIV care may lead to earlier ascertainment of comorbidities in health care clients in Khayelitsha, Cape Town

**DOI:** 10.1371/journal.pgph.0000031

**Published:** 2021-12-22

**Authors:** Richard Osei-Yeboah, Tsaone Tamuhla, Olina Ngwenya, Nicki Tiffin

**Affiliations:** 1 Division of Computational Biology, Integrative Biomedical Sciences Department, Faculty of Health Sciences, University of Cape Town, Cape Town, South Africa; 2 Wellcome Centre for Infectious Disease Research in Africa, Institute of Infectious Diseases and Molecular Medicine, University of Cape Town, Cape Town, South Africa; 3 Centre for Infectious Disease Epidemiology Research, School of Public Health and Family Medicine, University of Cape Town, Cape Town, South Africa; APHRC: African Population and Health Research Center, KENYA

## Abstract

Successful antiretroviral rollout in South Africa has greatly increased the health of the HIV-positive population, and morbidity and mortality in PLHIV can increasingly be attributed to comorbidities rather than HIV/AIDS directly. Understanding this disease burden can inform health care planning for a growing population of ageing PLHIV. Anonymized routine administrative health data were analysed for all adults who accessed public health care in 2016–2017 in Khayelitsha subdistrict (Cape Town, South Africa). Selected comorbidities and age of ascertainment for comorbidities were described for all HIV-positive and HIV-negative healthcare clients, as well as for a subset of women who accessed maternal care. There were 172 937 adult individuals with a median age of 37 (IQR:30–48) years in the virtual cohort, of whom 48% (83 162) were HIV-positive. Median age of ascertainment for each comorbidity was lower in HIV-positive compared to HIV-negative healthcare clients, except in the case of tuberculosis. A subset of women who previously accessed maternal care, however, showed much smaller differences in the median age of comorbidity ascertainment between the group of HIV-positive and HIV-negative health care clients, except in the case of chronic kidney disease (CKD). Both HIV-positive individuals and women who link to maternal care undergo routine point-of-care screening for common diseases at younger ages, and this analysis suggests that this may lead to earlier diagnosis of common comorbidities in these groups. Exceptions include CKD, in which age of ascertainment appears lower in PLHIV than HIV-negative groups in all analyses suggesting that age of disease onset may indeed be earlier; and tuberculosis for which age of incidence has previously been shown to vary according to HIV status.

## Introduction

South Africa bears the highest burden of the human immunodeficiency virus (HIV) epidemic in sub-Saharan Africa (SSA) [1] where about 7.2 million people are affected [2], and is considered the current epicentre of the HIV epidemic [3]. Antiretroviral therapy (ART) became available in 2004 but prior to the evolution of guidelines to the current “test and treat” policy in South Africa, many people living with HIV (PLHIV) did not reach older ages due to high mortality. Over the last decade, South Africa has expanded the ART and HIV prevention campaign by investing about $1.1 billion annually to run what is considered the largest program worldwide [4]. In 2014, Boulle et al., reported a rapid decline in mortality among HIV-infected patients with an increased duration on ART in South Africa and emphasized that though the rates may not be comparable to developed countries initially, after four years on ART they approach the rates observed in high-income countries where HIV is now treated as a chronic infection [5]. A decline in the percentage of acquired immunodeficiency syndrome (AIDS)-related mortalities from 42.1% in 2004 to 23.4% in 2019 [2], and the rising general life expectancy [2, 5] demonstrate the success of this ART programme. The prevalence of HIV in the population aged >50 years increased from 7.1% in 2012 to 12.5% in 2017 in South Africa [6].

The success of the ART intervention has resulted in more older people living with HIV, with improvements in their general quality of life, but in this ageing population other comorbidities still negatively affect mortality rate and life course outcome [7]. In addition to ongoing environmental and behavioural exposures that affect the whole population, comorbidities in PLHIV may also include HIV-related conditions such as HIV-induced persistent immunodeficiency, inflammation, and increased toxicity from longer durations of ART use [8], or HIV nephropathy [9]. Ageing also affects the immune system and the production and function of T cells, and these effects are worsened by HIV [10]. Some drugs included in standard regimens may also increase the risks of some non-communicable diseases (NCDs) [11]. A low progress in the care continuum is reported for PLHIV plus cardiometabolic conditions [12], and a significant proportion of mortalities in PLHIV may result from co-infections and NCDs such as malignancies, diabetes mellitus, hypertension, lipid disorders, and vascular diseases.

Recent studies outside Africa suggest higher prevalence and earlier incidence of comorbidities in PLHIV, especially those older than 50 years [13, 14], but more research is needed in SSA to see if similar trends may be seen [15]. Studies in 2015 and 2017 reported risks of—and increased—hospital admissions of PLHIV for AIDS-defining illnesses, renal and cardiovascular-related issues in Cape Town and other parts of South Africa [16–18], and the observations of hypertension, congestive cardiac failure, cancer and diabetes among PLHIV in Zimbabwe [19] suggest a growing burden of HIV comorbidities. Given the expanding ageing population of PLHIV within SSA, we need to understand the health needs of this population group now and in the future, as well as identifying potential drivers of comorbidities that may provide avenues for future interventions. In light of the current COVID-19 pandemic, it is more important than ever to understand the background of comorbidities and co-infections in a population at high risk of COVID-19 [20].

In this study, we analyze routine health data collected from a variety of public healthcare facilities including primary health care clinics, district level and tertiary hospitals across the Western Cape Province in South Africa. These data are used to describe ascertained comorbidities in a healthcare-seeking population from Khayelitsha, a high-density urban district in Cape Town. The Competition Commission reported that in 2018, approximately 83 per cent of the South African population who were mostly without medical insurance relied on public healthcare facilities and private healthcare facilities served the remaining 17 per cent with medical insurance [21–23]. Govender et al., report that among low-income patients in South Africa, affordability and convenience account for the top reasons influencing healthcare-seeking behaviour in public facilities whilst the quality of care accounts for the key reason for private healthcare facilities, and further observed a cycling behaviour between public and private sector clinics [24]. The subdistrict Khayelitsha, where this cohort originates, has a generally low-income population where we anticipate the majority of residents will access public health facilities.

We describe the median age of ascertainment for comorbidities in PLHIV and also for HIV-negative health care clients. In order to better understand the relationship between age of ascertainment and likely access to screening for common comorbidities, we compare the age of ascertainment for comorbidities in the subset of women accessing maternal care under the assumption that all women accessing maternal care are likely to receive screening for comorbidities at a younger age regardless of their HIV or other health status.

## Materials and methods

### Ethics

Ethics approval was obtained from the Human Research Ethics Committee of the Faculty of Health Sciences, University of Cape Town (HREC ref: 482/2019). A waiver for consent was granted because the data were anonymized and perturbed, and individuals could not be identified or re-identified from the data. A data access request was approved by the Health Impact Assessment Directorate at the Western Cape Department of Health, South Africa. There was no involvement of the public or patients because the data were accessed as an anonymized, perturbed dataset from routine data platforms without any interactions with individuals.

### Data source

The PHDC is a health information exchange facility that collates administrative health data for the Western Cape Province. Unique identifiers are used to link individuals to administrative health records [25], and facility visit, laboratory, and pharmacy data are updated daily for about 6.6 million people currently seeking care in public facilities in the Western Cape Province. Algorithms are used to infer disease episodes from combinations of pharmacy-dispensed drugs, laboratory test results, ICD-10 diagnosis codes, and facility encounter data. These algorithms are developed and tested in collaboration with clinicians who specialise in each condition. A data set was obtained from the Provincial Health Data Centre (PHDC), Western Cape Government Health Department, with longitudinal data ranging from 2007 to 2017. The median length of time for which individuals have available data is 8 years (IQR: 3.6–10 years). The study dataset was anonymised and perturbed prior to release, to prevent identification or re-identification of individuals. The electronic confirmation of disease diagnosis resulting from administrative health record linkage is referred to as “ascertainment” rather than diagnosis, as it is derived from the electronic records rather than from a diagnosis made by a clinician during consultation.

### Study population

All adults (≥18 years) who accessed public health facilities in the Khayelitsha subdistrict between 1 January 2016 and 31 December 2017, described as the ‘recruitment period’, were included in this study. Khayelitsha is a high-density, mixed informal/formal housing suburb in Cape Town, South Africa.

Descriptive statistics were generated for age, gender and burden of comorbidities in this study population. The comorbidities assessed were tuberculosis (TB)–using the age of ascertainment for first known episode, chronic obstructive pulmonary disease and/or asthma (COPD/Asthma), hypertension, diabetes, chronic kidney disease (CKD), cervical cancer, lung cancer, breast cancer, and mental health diagnoses. Cardiovascular disease was not included in this study because the PHDC algorithm to infer cardiovascular disease is not yet validated.

The age at ascertainment of each comorbidity in HIV-negative and HIV-positive subgroups of the total healthcare-seeking population was determined.

In addition to describing metrics for all healthcare seekers, age at ascertainment for each comorbidity was determined in a subset of all women who had ever accessed some form of pregnancy and/or maternal care. This subset was chosen to represent individuals who would have been linked to care independent of their HIV and general health status and are very likely to have undergone screening for common conditions as young adults. This subgroup was used to compare the age of ascertainment of comorbidities in HIV-positive and HIV-negative strata, in order to indicate whether earlier linkage to care might lead to earlier ascertainment of these comorbidities. The significance of difference between median ages at ascertainment was calculated using Wilcoxon sum ranked tests, and the significance of difference in proportions of comorbidities between PLHIV and HIV-negative groups in this subset was calculated using Fisher’s exact test.

Multivariate logistic regression was used to assess the likelihood of individuals seeking healthcare for each condition to also present with HIV and other comorbidities. Each comorbidity was independently assessed as an outcome/dependent variable with independent variables age, sex, HIV, and other comorbidities. This approach was used to accommodate known bias in the dataset.

Data analyses were done using R Software (version 3.6.0) and RStudio (version 1.1.447); Graphical representations of age distributions at start of recruitment period for HIV-negative and -positive population, sex, age at ascertainment of HIV, and comorbidities distributions by age, HIV status, and sex were generated using the ggplot2 package in RStudio version 1.1.447.

### Patient and public involvement

The participants in this study were healthcare seekers who visited public health facilities and generated at least one electronic health record. Retrospective data for this population spanned about 8 years. Inclusion in the study was restricted to healthcare clients who accessed care between 2016 and 2017 but included their complete retrospective data. The study questions were designed to explore the common comorbidities among these healthcare clients who seek care from public facilities. A waiver for participants’ consent was granted because the data were obtained directly from digital routine health data in the PHDC and were anonymized and perturbed to prevent re-identification of participants.

## Results

### Study population characteristics

The total study population was 172 937 healthcare seekers, with a median age of 37 years (IQR:30–48 years), of which 125 468 (73%) were females. There were 83 162 HIV-positive individuals—48% of the total healthcare-seeking population. There were 59 164 HIV-positive females, representing 71% of all PLHIV. There were 67 499 women with evidence of previous access to maternal care, more than half (54%) of all female healthcare seekers. Of those who had accessed maternal care, 29 828 (44.2%) were living with HIV. About 46.6% of PLHIV were seeking care for the additional comorbidities investigated in this study compared to 61.5% of individuals without HIV (Table 1).

**Table 1:** Demographic and baseline characteristics of healthcare seekers.

Healthcare seeking population	Total	HIV- (%)	HIV+ (%)
	172 937	89 775 (52%)	83 162 (48%)
**Female**	125 468 (72.6%)	66 304 (73.9%)	59 164 (71.1%)
**Has accessed maternal care**	67 499 (53.8% *)	37 671 (55.8%**)	29 828 (44.2%**)
**No comorbidity**	78 990 (45.7%)	34 575 (38.5%)	44 415 (53.4%)
**1 comorbidity**	65 207 (37.7%)	36 701 (40.9%)	28 506 (34.3%)
**2 comorbidities**	21 514 (12.4%)	13 674 (15.2%)	7 840 (9.4%)
**≥3 comorbidities**	7 226 (4.2%)	4 825 (5.4%)	2 401 (2.9%)

*Proportion of females **Proportion of those who accessed maternal care.

**Fig 1:**
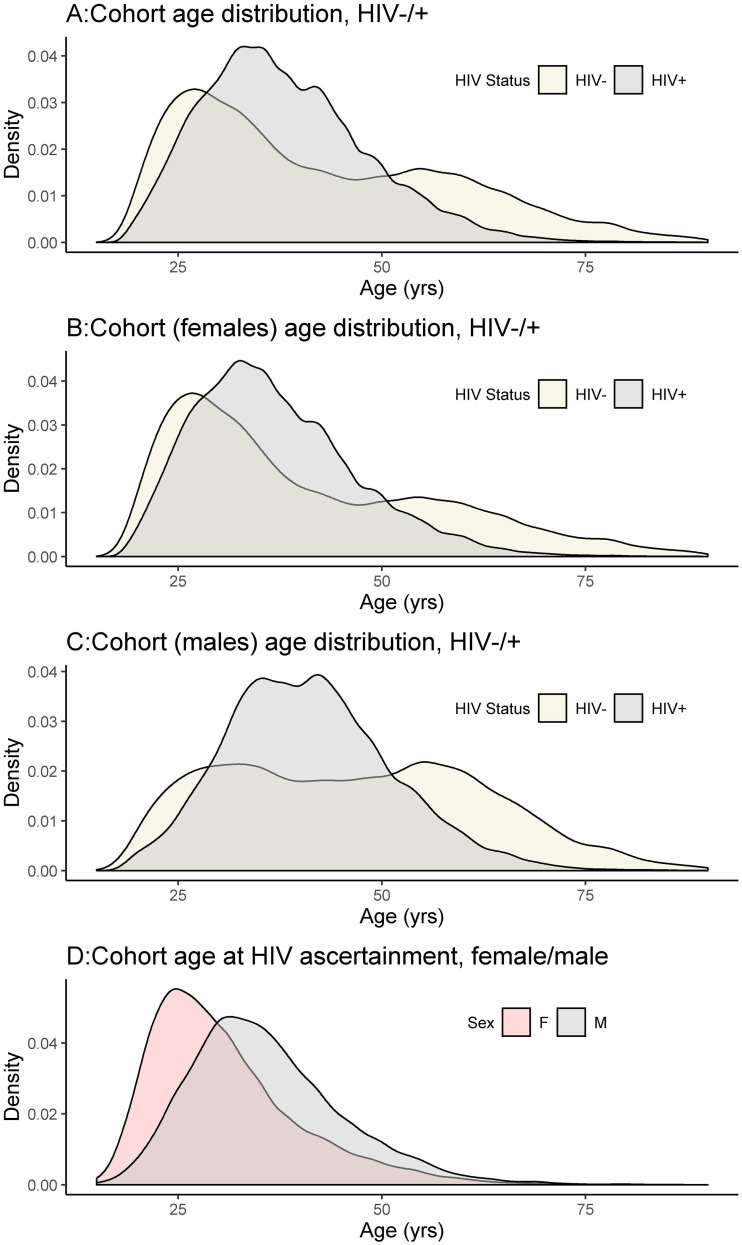
Age distributions at the beginning of the recruitment period. Age in years is shown for the beginning of the recruitment period, subgroups shown are HIV status and sex.

The age distribution assessed at the beginning of the recruitment period for females and males, as well as HIV-negative and HIV-positive healthcare seekers, shows a non-uniform distribution: there were more women than men in this cohort of healthcare seekers, with more HIV-positive individuals in the younger age groups. More women were living with HIV at younger ages than men and their HIV-positive status was ascertained at earlier ages than men (Fig 1).

### The burden of comorbidities and age of ascertainment in HIV-positive and HIV-negative healthcare seekers

The proportion of HIV-positive and HIV-negative individuals seeking care in public health facilities with the assessed comorbidities were: have ever had tuberculosis (21.4%), chronic obstructive pulmonary diseases or asthma (7.4%), hypertension (26.4%), diabetes (9.7%), chronic kidney disease (2.4%), cervical cancer (0.9%), lung cancer (0.5%), breast cancer (0.4%), and mental health conditions (7.2%), (Table 2). The median age (IQR) of HIV ascertainment differs in females and males at 35 years (IQR:30–43 years) and 40 years (IQR: 34–47 years) respectively *(p* <0.001). Except for tuberculosis, all comorbidities were ascertained earlier among people living with HIV, with large differences in the age of ascertainment seen between HIV-negative and HIV-positive healthcare seekers (Table 2).

**Table 2:** Comorbidities counts and median age (in years) at ascertainment in HIV-negative vs HIV-positive for all healthcare seekers.

Condition	Healthcare seeking population		HIV-negative n = 89 775		HIV-positive n = 83 162	
	Count (%) n = 172 937	Age at ascertainment (IQR)	Count (%)	Age at ascertainment (IQR)	Count (%)	Age at ascertainment (IQR)
Tuberculosis	36 837 (21.3%)	34 (27–42)	11 298 (12.6%)	33 (24–48)	25 539 (30.7%)	34 (28–41)
COPD/Asthma	12 820 (7.4%)	45 (32–56)	8 477 (9.4%)	50 (33–60)	4 343 (5.2%)	39 (32–48)
Hypertension	45 691 (26.4%)	49 (40–58)	34 090 (38%)	52 (43–60)	11 601 (14%)	43 (36–50)
Diabetes	16 979 (9.8%)	51 (41–59)	13 561 (15.1%)	52 (44–61)	3 418 (4.1%)	44 (36–51)
Chronic Kidney Disease	4 179 (2.4%)	57 (48–67)	2 833 (3.2%)	62 (55–71)	1 346 (1.6%)	46 (38–55)
Cervical Cancer*	1 180 (0.9%)	38 (32–47)	294 (0.4%)	52 (30–61)	886 (1.5%)	36 (31–42)
Lung Cancer	784 (0.5%)	47 (34–59)	443 (0.5%)	56 (40–65)	341 (0.4%)	39 (31–49)
Breast Cancer	691 (0.4%)	44 (33–54)	458 (0.5%)	47 (34–57)	233 (0.3%)	40 (33–46)
Mental Health Condition	12 512 (7.2%)	37 (27–50)	8 279 (9.2%)	39 (26–54)	4 233 (5.1%)	36 (29–45)

*Proportions for cervical cancer were calculated for the female population only.

Within the subset of women who have ever accessed maternal care, however, the differences in the median ages at ascertainment for each comorbidity were much smaller compared to the whole population of healthcare seekers, except in the case of chronic kidney disease where the median age of ascertainment was approximately 5.5 years earlier in HIV-positive women (*p*<0.001), and tuberculosis where the median age of ascertainment was approximately 5 years later in HIV-positive women (*p*<0.001) (Table 3). The percentage of HIV-negative and HIV-positive women presenting with each comorbidity in this subset are shown (Table 3). HIV-positive women were more likely to present with tuberculosis (OR:6.78, 95% CI: 6.40, 7.18); CKD (OR:3.48, 95% CI:2.67, 4.58); cervical cancer (OR:9.47, 95% CI: 7.22,12.60); lung cancer (2.39, 95% CI:1.65, 3.5) and mental health conditions (OR:1.41, 95% CI:1.30, 1.53). They were less likely to present with diabetes (OR:0.69, 95% CI:0.64, 0.75).

**Table 3:** Comorbidity counts (%) with Odds Ratio (95% CI), and age at ascertainments of comorbidities (with Wilcoxon Rank Sum p-value), for HIV-negative and HIV-positive women who have accessed maternal care (IQR: Interquartile range).

Condition	Women who accessed maternal care		HIV-negative women (n = 37 671)		HIV-positive women (n = 29 828)		OR (C.I) Comorbidity count	P-value Ascertainment age
	Count (%) n = 67 499	Ascertainment age (IQR)	Count (%)	Ascertainment age (IQR)	Count (%)	Ascertainment age (IQR)		
Tuberculosis	8 416 (12.5%)	29 (24–34)	1 583 (4.2%)	24 (21–30)	6 833 (22.9%)	29 (25–34)	6.78 (6.40–7.18)	<0.001
COPD/Asthma	2 587 (3.8%)	32 (36–38)	1 225 (3.3%)	31 (25–38)	1 362 (4.6%)	33 (28–38)	1.42 (1.31–1.54)	<0.001
Hypertension	7 475 (11.1%)	36 (30–41)	4 202 (11.2%)	36 (30–42)	3 273 (11%)	35 (30–40)	0.98 (0.93–1.03)	0.008
Diabetes	2 434 (3.6%)	35 (29–41)	1 565 (4.2%)	36 (30–41)	869 (2.9%)	35 (29–40)	0.69 (0.64–0.75)	0.05
Chronic Kidney Disease	292 (0.43%)	39 (33–45)	78 (0.21%)	43 (36–47)	214 (0.72%)	37.5 (32–44)	3.48 (2.67–4.58)	<0.001
Cervical Cancer	504 (0.75%)	33 (29–38)	60 (0.16%)	34 (31–39∙5)	444 (1.5%)	33 (29–38)	9.47 (7.22–12.60)	0.05
Lung Cancer	130 (0.19%)	31 (26–37)	45 (0.12%)	31 (26–41)	85 (0.28%)	32 (26–36)	2.39 (1.65–3.51)	0.79
Breast Cancer	212 (0.31%)	33.5 (27.8–41)	120 (0.32%)	30∙5 (26–41)	92 (0.31%)	35.5 (29–40)	0.97 (0.73–1.28)	0.05
Mental Health Condition	2 480 (3.7%)	32 (26–38)	1 178 (3.1%)	31 (25–38)	1 302 (4.4%)	32 (27–38)	1.41(1.30–1.53)	<0.001

### Distribution of ascertainment age for comorbidities in HIV-negative and HIV-positive healthcare seekers

**Fig 2:**
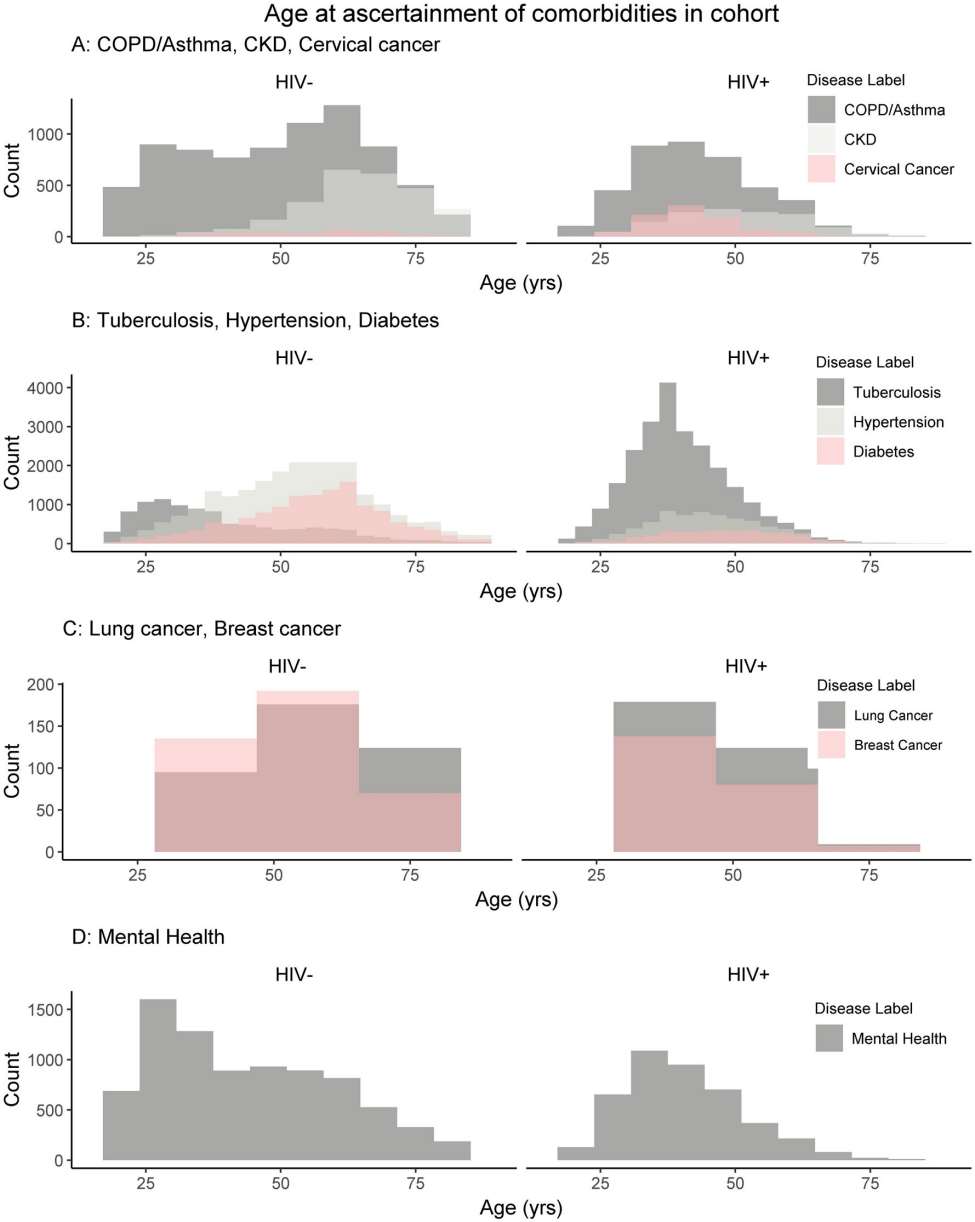
Age at ascertainment for comorbidities for HIV-positive and HIV-negative health care seekers. The absolute counts of comorbidities are shown, grouped by count range for optimal display. A. COPD/Asthma, CKD, and cervical cancer. B. Tuberculosis, hypertension, and diabetes. C. Lung cancer and breast cancer. D. Mental health condition.

**Fig 3:**
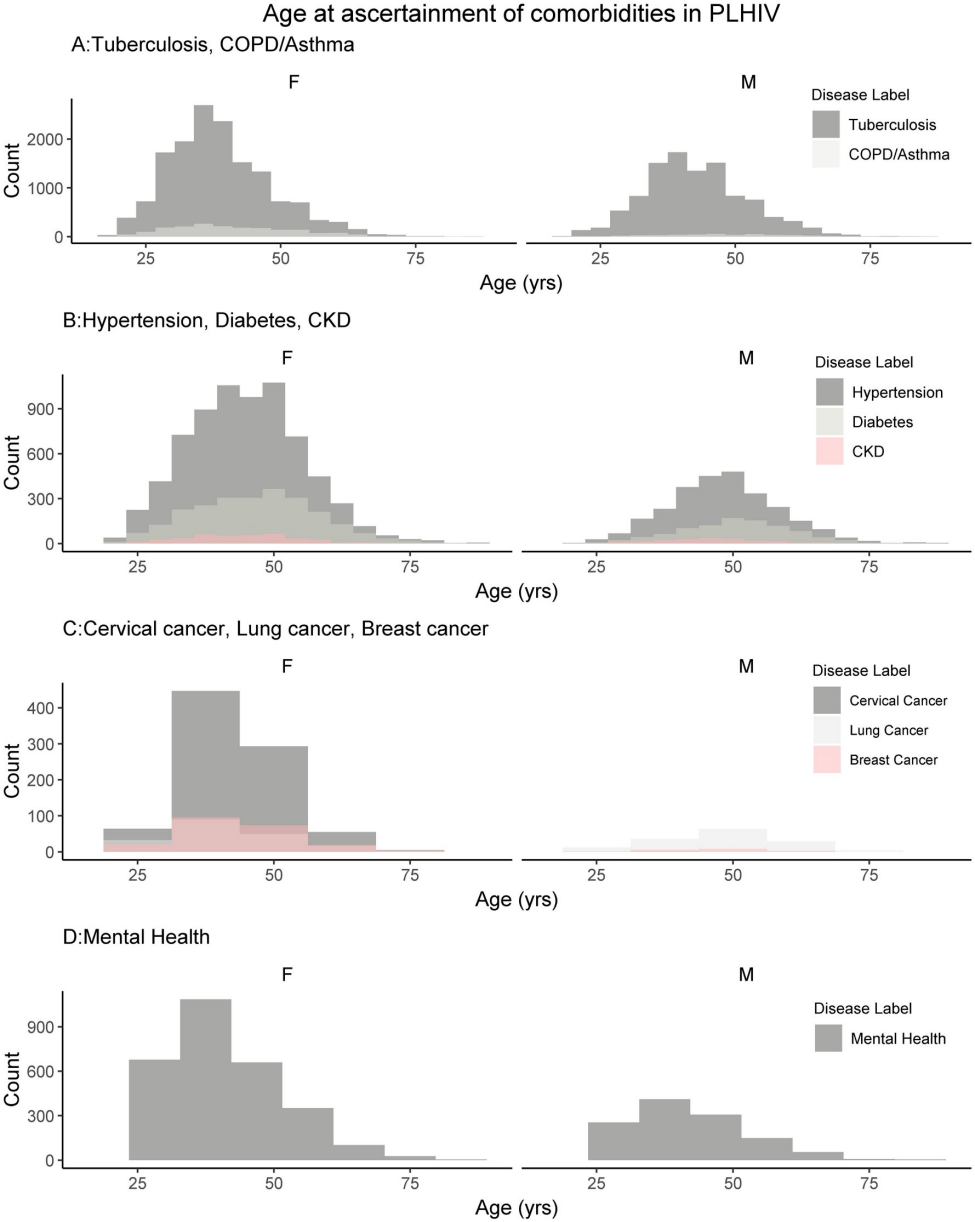
Age at ascertainment of comorbidities in HIV-positive healthcare seekers by sex. The absolute counts of comorbidities are shown, grouped by count range for optimal display. A. COPD/Asthma, CKD, and cervical cancer. B. Tuberculosis, hypertension, and diabetes. C. Lung cancer and breast cancer. D. Mental health condition.

The distributions of age at ascertainment for the comorbidities assayed are shown in Fig 2 for both HIV-negative and -positive groups and Fig 3 for PLHIV. Generally, in the HIV-positive healthcare seekers, all comorbidities are ascertained across a narrower range of ages, whilst ascertainment of comorbidities in HIV-negative healthcare seekers show a wider age range. There is a drop off in ascertainment of comorbidities in HIV-positive individuals at older ages.

### HIV status of individuals presenting with common conditions and other comorbidities

Multivariate logistic regression analysis shows the likelihood of having HIV and other comorbidities in patients presenting with each condition. As expected, the odds of having all conditions increased with age, calculated per 5-year increments. In line with existing studies, within the whole study population, people who seek care for a first episode of TB are 2.74 (95% C.I:2.66, 2.81) times more likely to be HIV-positive than HIV-negative. People presenting with CKD are 1.67 (95% CI:1.54, 1.82) times likely to be HIV-positive and those presenting with cervical cancer are 4.90 (95% CI:4.22, 5.71) times more likely to be HIV-positive. The complete data are shown in S1 Table.

The subset of women who ever accessed maternal care was used to estimate the contribution of HIV to the likelihood of having each comorbidity when adjusting for age and the co-occurrence of other comorbidities simultaneously, using multivariate logistic regression analysis. This subset was analysed in order to ameliorate the impact of the bias in the composition of the whole study population, using access to maternal care as a proxy for linkage to care at a younger age without necessarily presenting with ill health. The results presented in Table 4, show independent analyses with each assayed comorbidity modelled as the outcome, and the contribution of HIV when adjusted for the other comorbidities. In particular, within the subset of those who have ever accessed maternal care, women who ever had tuberculosis are 6.24 (95% CI: 5.89, 6.61) times more likely than those without tuberculosis to also present with HIV; those with COPD/Asthma are 1.14 (95% CI:1.04, 1.24) and 1.76 (95% CI:1.58, 1.95) times more likely to have had HIV and TB, respectively; and maternal care seekers presenting with cervical cancer are 7.41 (95% CI:5.67, 9.7) times more likely to be HIV-positive compared to those without cervical cancer.

**Table 4:** Multivariate logistic regression for women who have previously accessed maternal care; showing odds ratio (95% Confidence interval). Odds ratio for age is shown per 5-year increments.

OUTCOMES	INDEPENDENT VARIABLES (OR [95% C.I])										
	HIV	TB	COPD/Asthma	Hypertension	Diabetes	CKD	Cervical cancer	Lung cancer	Breast cancer	Mental Health	Age 5 yr. increment
Tuberculosis	6.24 (5.89,6.61)	-	1.74 (1.56,1.92)	0.64 (0.58,0.69)	1.18 (1.04,1.34)	2.82 (2.18,3.65)	1.33 (1.08,1.62)	3.38 (2.29,4.95)	1.28 (0.86,1.85)	1.88 (1.69,2.09)	1.15 (1.12,1.16)
COPD/Asthma	1.14 (1.04,1.24)	1.76 (1.58,1.95)	-	1.80 (1.62,2.00)	1.14 (0.96,1.35)	1.55 (1.07,2.19)	0.97 (0.65,1.38)	1.65 (0.85,2.90)	1.27 (0.73,2.06)	2.02 (1.74,2.33)	1.20 (1.18,1.24)
Hypertension	0.83 (0.79,0.88)	0.65 (0.59,0.70)	1.78 (1.60,1.98)	-	3.70 (3.37,4.07)	3.32 (2.52,4.36)	1.02 (0.80,1.30)	1.12 (0.66,1.82)	1.46 (1.02,2.06)	1.68 (1.50,1.88)	1.85 (1.82,1.89)
Diabetes	0.56 (0.51,0.61)	1.26 (1.10,1.43)	1.08 (0.91,1.29)	3.66 (3.32,4.03)	-	1.98 (1.42,2.71)	1.32 (0.91,1.87)	0.72 (0.25,1.63)	0.61 (0.30,1.10)	1.33 (1.12,1.58)	1.53 (1.50,1.58)
CKD	2.74 (1.86,3.30)	3.01 (2.33,3.90)	1.57 (1.08,2.23)	3.48 (2.66,4.54)	2.25 (1.62,3.08)	-	1.91 (1.00,3.32)	1.74 (2.79,5.78)	0.24 (0.01,1.26	1.81 (1.24,2.57)	1.70 (1.57,1.85)
Cervical cancer	7.41 (5.67,9.7)	1.33 (1.09,1.63)	0.95 (0.64,1.36)	1.03 (0.80,1.31)	1.31 (0.90,1.85)	1.52 (0.80,2.66)	-	3.42 (1.30,7.42)	1.10 (0.32,2.79)	1.77 (1.28,2.39)	1.63 (1.53,1.73)
Lung cancer	1.37 (0.92,1.07)	3.42 (2.31,5.03)	1.59 (0.82,2.81)	1.14 (0.67,1.85)	0.67 (0.23,1.55)	1.18 (0.19,3.95)	3.13 (1.20,6.71)	-	3.90 (0.92,10.9)	2.29 (1.28,3.83)	1.25 (1.11,1.41)
Breast cancer	0.73 (0.54,1.09)	1.41 (0.95,2.04)	1.28 (0.74,2.08)	1.47 (1.03,2.08)	0.62 (0.31,1.11)	0.34 (0.12,1.55)	1.36 (0.41,3.320	3.86 (0.92,10.9)	-	5.61 (3.96,7.80)	1.42 (1.30,1.56)
Mental Health Condition	1.11 (1.02,1.22)	1.90 (1.71,2.10)	2.00 (1.73,2.32)	1.71 (1.52,1.90)	1.35 (1.14,1.60)	1.63 (1.12,2.32)	1.76 (1.27,2.37)	2.34 (1.30,3.91)	5.68 (4.00,7.91)	-	1.16 (1.13,1.19)

In general, hypertension, diabetes and CKD had increased odds of co-occurring, as expected (Table 4). When adjusting for these comorbidities, the impact of HIV could be more clearly determined. Individuals with hypertension from this subset are 17% less likely to present with HIV (OR: 0.83, 95% CI:0.79,0.88), those with diabetes are 44% less likely to present with HIV (OR:0.56, 95% CI:0.51,0.61), and those with CKD are 2.74 (95%CI:1.86, 3.30) times more likely to present with HIV. There is no significant difference in HIV presentation between those with and without lung cancer or breast cancer. Finally, women who accessed maternal care and have mental health conditions are 11% more likely to present with HIV (OR:1.11, 95% CI: 1.02, 1.22) as well as other comorbidities compared to those without mental health conditions.

## Discussion

Our results reveal that PLHIV in Khayelitsha, Cape Town are seeking care for multiple chronic comorbidities in addition to co-infection with tuberculosis. Analysis of the healthcare client population in this study shows earlier ascertainment of most chronic comorbidities in PLHIV. Whilst this could be due to generally earlier incidence of comorbidities in the HIV-positive population, it could also reflect an earlier diagnosis of comorbidities in those with frequent access to health care and earlier screening due to HIV treatment visits: ascertainment of comorbidities might occur later in people who do not normally access health care frequently and therefore only receive a diagnosis when comorbidities are sufficiently advanced to present with symptoms. Statistical metrics were not used to directly compare the prevalence of comorbidities in HIV-positive and HIV-negative subsets of the overall study population–people seeking healthcare—due to the known bias in this dataset which is enriched for people who are already ill or have frequent healthcare-seeking behaviour due to existing chronic conditions such as HIV. Bias also results from young healthy women attending healthcare facilities for contraceptive or maternal health services, whilst young, healthy men seldom access health care services. The general populations of healthcare seekers who are HIV-positive and HIV-negative are not directly comparable, accordingly.

To further explore ascertainment of comorbidities in an unbiased subset of this population, we analysed data for a subset of women who have previously accessed maternal care, under the assumption they are likely to have been screened for common comorbidities such as hypertension, diabetes, and kidney disease during their pregnancy. This provided a proxy dataset for individuals who have been screened at a younger age even in the absence of known health conditions or symptoms. The much smaller differences in ages at ascertainment of most comorbidities among women seeking maternal care in both HIV-negative and HIV-positive groups that we identified suggest that frequent access to healthcare may result in earlier ascertainment of these comorbidities, rather than there being generally earlier incidence in the HIV-positive population. In all comorbidities assayed where significant differences were identified in the age of ascertainment of this group, the difference is in the range of only 1–2 years—with the notable exception of CKD which occurs an average of 5.5 years earlier in HIV positive women, in line with existing studies on HIV Nephropathy [26, 27]. For tuberculosis, the median age of ascertainment is approximately 5 years higher in PLHIV, and we believe this may reflect the difference in age distribution for tuberculosis incidence in HIV-negative and HIV-positive individuals: for those without HIV, tuberculosis risk is high in young adults but decreases rapidly at older ages [28] whereas tuberculosis risk in PLHIV remains elevated throughout adulthood, leading to a shift to an older *median* age of first tuberculosis ascertainment. We anticipate that data for cardiovascular disease (CVD) in this population may also show earlier occurrence in PLHIV, based on prior studies [29], and we will conduct a similar analysis for CVD when these data are available. Multivariate analysis shows that in women, having tuberculosis or cervical cancer is highly associated with being HIV-positive. Both arise from infectious agents and are classified as HIV-related conditions [30].

We recognize the bias in our dataset due to imbalances in the sectors of the population commonly seeking healthcare, and the exclusion of many healthy individuals (especially young men) who do not frequently attend healthcare facilities. Bias in the data means that direct comparisons of comorbidity prevalence could not be made between the total HIV-positive and HIV-negative study groups. Several sources of bias exist: healthy HIV-negative individuals without comorbidities are under-represented in people commonly seeking healthcare in public facilities; HIV-negative health care clients are likely to be seeking healthcare because they are ill with other conditions, so the HIV-negative group in the study population is enriched for other comorbidities; and individuals with conditions requiring frequent medication–especially HIV medication–are more likely to visit a facility during the recruitment period and subsequently be included in the dataset, so the study population is further enriched for HIV-positive individuals. In the subset analysis, women who have accessed maternal health services previously were selected to represent a subgroup of individuals who have accessed care and received screening for common comorbidities at a younger age, regardless of their HIV- or general health status, thus providing a less-biased subset for additional analyses. Whilst this analysis can address the bias resulting from differences in accessing health care services, some of the limitations of the maternal subset analysis include our inability to assess comorbidities occurring more commonly in men, or much older women. In addition, it is possible that pregnant women living with HIV may have more rigorous screening and antenatal care which may lead to more frequent ascertainment of existing conditions that for HIV-negative pregnant. As we collect more data about this cohort over time, we will also be able to analyse evolving comorbidity profiles as the maternal cohort ages.

Individuals visiting healthcare facilities who are not seeking care for HIV are more likely to be accessing care for one or more other comorbidities. This explains the high prevalence of these comorbidities in health care clients who are HIV-negative and does not accurately represent the prevalence of those comorbidities in the general HIV-negative population, many of whom may not be currently in active care.

For these reasons, we have not attempted to compare the estimated prevalence of comorbidities in PLHIV with those who are HIV-negative in this study population, and we did not use HIV status as an outcome for multivariate regression analysis. We have used the maternal subset as a proxy for a more balanced analysis, based on the assumption that women who access maternal health care do so at a relatively younger age regardless of their HIV status or other health conditions. Because of the time frame for which retrospective data are available, the subset who have ever accessed maternal care had a lower maximum age than the whole group (S1 Fig). The analysis of the maternal subset clearly cannot, however, be used to understand sex differences in the healthcare-seeking population.

Differences between female and male demographics of healthcare clients may also reflect to some extent contraceptive and maternal care access by women who are not experiencing health issues or poor health, and this group of health care clients contributes to the relatively high proportion of younger women without HIV presenting with no comorbidities in the data set. We do not see a similar proportion of young, healthy males reflected in this dataset, accordingly. Prior studies also suggest that women are more likely to have frequent healthcare-seeking behaviours than men which may also contribute to the higher numbers of women in this study [31], and frequent access to health care plays a pivotal role in the ascertainment of HIV among both younger and older women [32]. The later ascertainment of HIV among men compared to women could be a result of men only presenting to facilities when they are already ill [33], and health promotion and encouraging healthcare-seeking behaviour are key in ensuring early detection of HIV in this sector of the population [32].

A rapid fall-off in numbers of HIV-positive people aged over 60 years at recruitment is because prior to ART rollout in 2004, there was high mortality in PLHIV [34] and there are few people who were infected prior to 2004 and have now survived beyond 60 years of age. As the HIV-positive population now ages, however, the rising challenge of NCDs among ageing HIV-infected persons indicates that disease-specific care delivery for PLHIV may need to become more integrated and holistic to ensure that comorbidities in these patients receive the necessary attention.

## Conclusion

Ascertainment of comorbidities relies on screening, which is influenced by healthcare-seeking behaviours. Our analysis suggests that when women link to maternal care, or PLHIV link to HIV care, which both include point-of-care screening, they have earlier ascertainment of common conditions. This may be a more likely explanation for earlier age of ascertainment of comorbidities in PLHIV than in HIV-negative individuals, rather than earlier disease onset. If this holds true in the wider population, it would suggest that earlier screening, in general, could lead to earlier ascertainment of common comorbidities–in turn leading to earlier linkage to care and better patient outcomes. Our data also suggest that as PLHIV age, their comorbidities curve will also widen toward the older ages and share similarities with the distribution of comorbidities in HIV-negative healthcare seekers, increasing the burden on existing healthcare facilities. Careful planning can ensure that this ageing population has sufficient access to healthcare for HIV and comorbidities, into the future.

## Supporting information

S1 Fig Age distribution of women in maternal subgroup, women in non-maternal subgroup, and subgroup with equivalent age range. Legend: Age (yrs.) along the x-axis is the distribution of age at the beginning of the recruitment period in HIV-negative and HIV-positive groups. A. Women who have ever accessed maternal care. B. Non-maternal women from the general healthcare-seeking population with an equivalent age range. C. The general healthcare-seeking population with an equivalent age range.
(PDF)

S2 Fig Age at ascertainment of comorbidities in HIV-negative individuals. The absolute counts of comorbidities are shown, grouped by count range for optimal display. A. Tuberculosis and COPD/Asthma. B. Hypertension, Diabetes and CKD. C. Breast cancer, Lung cancer and Cervical cancer. D. Mental health condition.
(PDF)

S1 Table Multivariate logistic regression of healthcare-seeking population; Odds ratio (95% Confidence interval). Odds ratio for age is shown in 5-year increments.
(PDF)

## Abbreviations

AIDS: Acquired Immune Deficiency Syndrome
ART: Antiretroviral Therapy
CI: Confidence Interval
CKD: Chronic Kidney Disease
COPD: Chronic Obstructive Pulmonary Disease
CVD: Cardiovascular Disease
HIV: Human Immunodeficiency Virus
ICD-10: International Classification of Diseases-10
IQR: Interquartile Range
NCD: Non-Communicable Disease
OR: Odds Ratio
PHDC: Provincial Health Data Centre
PLHIV: People Living with HIV
SSA: sub-Saharan Africa
TB: Tuberculosis
