# Supplementary material for: Accessing HIV care may lead to earlier ascertainment of comorbidities in health care clients in Khayelitsha, Cape Town
Source: PLOS Glob Public Health. 2021 Dec 22;1(12):e0000031. doi: 10.1371/journal.pgph.0000031 (PMC10021146; doi:10.1371/journal.pgph.0000031)
Supplement: S1 Fig — Legend: Age (yrs.) along the x-axis is the distribution of age at the beginning of the recruitment period in HIV-negative and HIV-positive groups. A. Women who have ever accessed maternal care. B. Non-maternal women from the general healthcare-seeking population with an equivalent age range. C. The general healthcare-seeking population with an equivalent age range. (PDF) [file pgph.0000031.s001.pdf]

Osei-Yeboah et al. 2021 Supporting Information File S1 Fig

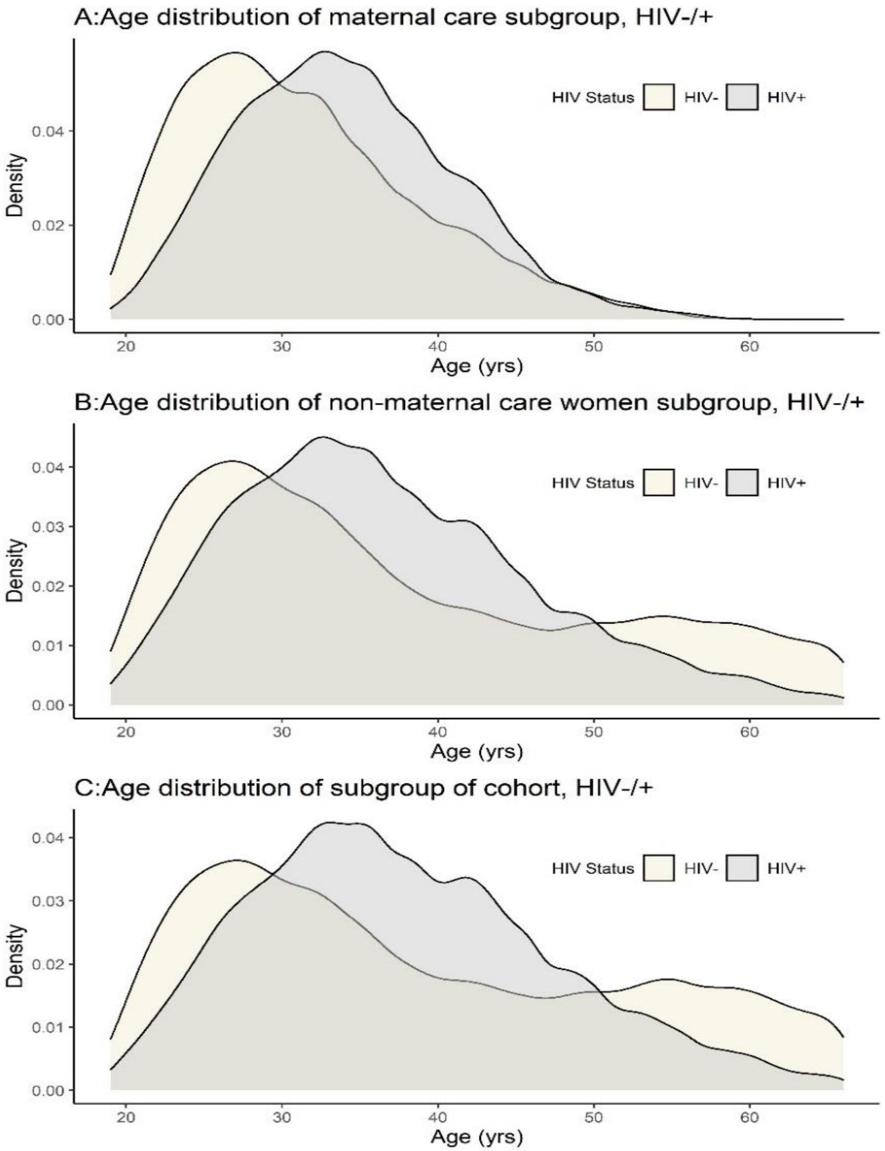

Supporting Information

S1 Fig: Age distribution of women in maternal subgroup, women in non- maternal subgroup, and subgroup with equivalent age range.

Legend: Age (yrs.) along the x-axis is the distribution of age at the beginning of the recruitment period in HIV-negative and HIV-positive groups. A. Women who have ever accessed maternal care. B. Non-maternal women from the general healthcare-seeking population with an equivalent age range. C. The general healthcare-seeking population with an equivalent age range.
