# Supplementary material for: Accessing HIV care may lead to earlier ascertainment of comorbidities in health care clients in Khayelitsha, Cape Town
Source: PLOS Glob Public Health. 2021 Dec 22;1(12):e0000031. doi: 10.1371/journal.pgph.0000031 (PMC10021146; doi:10.1371/journal.pgph.0000031)
Supplement: S2 Fig — The absolute counts of comorbidities are shown, grouped by count range for optimal display. A. Tuberculosis and COPD/Asthma. B. Hypertension, Diabetes and CKD. C. Breast cancer, Lung cancer and Cervical cancer. D. Mental health condition. (PDF) [file pgph.0000031.s002.pdf]

Osei-Yeboah et al. 2021 Supporting Information File S2 Fig

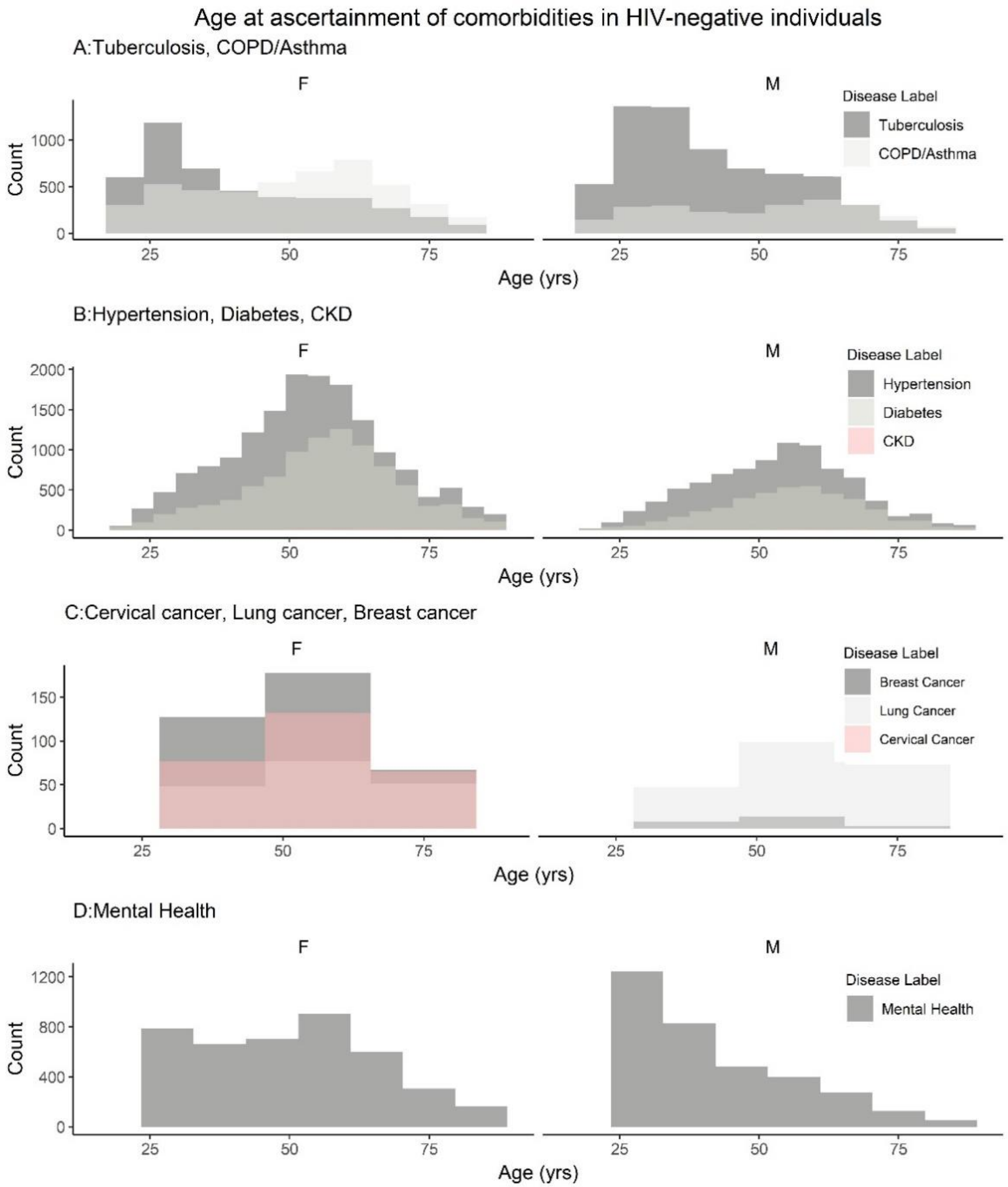

Supporting Information

S2 Fig: Age at ascertainment of comorbidities in HIV-negative individuals.

Legend: Age (yrs.) along the x-axis is the distribution of age at the beginning of the recruitment period for F (Females) and M (Males). The absolute counts of comorbidities are shown, grouped by count range for optimal display along y-axis. A. Tuberculosis and COPD/Asthma. B. Hypertension, Diabetes and CKD. C. Breast cancer, Lung cancer and Cervical cancer. D. Mental health condition
