# Supplementary material for: Accessing HIV care may lead to earlier ascertainment of comorbidities in health care clients in Khayelitsha, Cape Town
Source: PLOS Glob Public Health. 2021 Dec 22;1(12):e0000031. doi: 10.1371/journal.pgph.0000031 (PMC10021146; doi:10.1371/journal.pgph.0000031)
Supplement: S1 Table — Odds ratio for age is shown in 5-year increments. (PDF) [file pgph.0000031.s003.pdf]

Osei-Yeboah et al. 2021. Supporting Information File S1 Table

S1 Table: Multivariate logistic regression of healthcare-seeking population; Odds ratio (95% Confidence interval)

Odds ratio for age is shown for 5-year increments

**OUTCOMES**	**INDEPENDENT VARIABLES (OR [95% C.I])**											
	HIV	Tuberculosis	COPD/Asthma	Hypertension	Diabetes	CKD	Cervical cancer	Lung cancer	Breast cancer	Mental health condition	Age 5 yr incr.	Sex M
Tuberculosis	2.74 (2.66,2.81)	-	1.39 (1.33,1.46)	0.41 (0.39,0.43)	0.75 (0.72,0.79)	1.50 (1.38,1.63)	1.45 (1.27,1.67)	3.21 (2.76,3.75)	0.94 (0.75,1.16)	0.98 (0.93,1.03)	1.08 (1.07,1.08)	3.04 (2.96,3.12)
COPD/Asthma	0.61 (0.59,0.64)	1.43 (1.36,1.50)	-	1.10 (1.05,1.15)	0.79 (0.75,0.84)	1.04 (0.96,1.15)	1.02 (0.81,1.26)	2.45 (2.06,2.91)	1.04 (0.80,1.37)	1.20 (1.12,1.27)	1.20 (1.18,1.20)	1.10 (1.06,1.15)
Hypertension	0.40 (0.38,0.41)	0.43 (0.41,0.45)	1.00 (0.96,1.05)	-	1.66 (1.59,1.73)	1.81 (1.66,1.99)	1.14 (0.97,1.32)	0.51 (0.42,0.62	0.84 (0.69,1.02)	0.82 (0.77,0.86)	1.63 (1.61,1.64)	1.10 (1.08,1.14)
Diabetes	0.43 (0.41,0.45)	0.85 (0.80,0.89)	0.77 (0.73,0.82)	2.02 (1.94,2.11)	-	2.43 (2.26,2.62)	1.14 (0.93,1.38)	0.77 (0.61,0.97)	0.87 (0.68,1.09)	0.98 (0.93,1.05)	1.28 (1.27,1.29)	0.99 (0.96,1.03)
CKD	1.67 (1.54,1.82)	1.61 (1.48,1.75)	1.08 0.98,1.19)	2.40 (2.21,2.60)	2.94 (2.75,3.16)	-	1.76 (1.33,2.30)	0.82 (0.56,1.16)	1.08 (0.72,1.55)	1.57 (1.42,1.73)	1.42 (1.40,1.44)	1.00 (0.94,1.08)
Cervical cancer	4.90 (4.22,5.71)	1.24 (1.08,1.42)	0.87 (0.60,1.08)	1.01 (0.87,1.18)	0.98 (0.80,1.19)	1.23 (0.92,1.61)	-	2.54 (1.43,4.18)	2.23 (1.35,3.49)	1.95 (1.63,2.32)	1.24 (1.21,1.27)	0.001 (0.00,0.01
Lung cancer	0.79 (0.67,0.93)	3.44 (2.95,4.02)	2.49 (2.09,2.95)	0.68 (0.57,0.81)	0.85 (0.68,1.07)	0.85 (0.59,1.20)	2.91 (1.65,4.74)	-	3.94 (2.21,6.47)	1.78 (1.44,2.17)	1.24 (1.21,1.27)	1.65 (1.42,1.92)
Breast cancer	0.71 (0.60,0.85)	0.94 (0.75,1.16)	1.00 (0.77,1.28)	0.87 (0.72,1.04)	0.82 (0.65,1.03)	0.86 (0.58,1.23)	2.42 (1.46,3.76)	3.72 (2.08,6.15)	-	4.06 (3.39,4.84)	1.18 (1.15,1.21)	0.14 (0.11,0.20)
Mental health condition	0.52 (0.50,0.54)	0.94 (0.90,1.00)	1.17 (1.09,1.24)	0.82 (0.78,0.86)	0.96 (0.90,1.02)	1.67 (1.52,1.84)	2.66 (2.22,3.15)	1.61 (1.30,1.96)	4.44 (3.72,5.29)	-	1.04 (1.03,1.05)	1.95 (1.87,2.03)
